# The Physician’s Visiting List for 1897

**Published:** 1897-01

**Authors:** 


### BOOK NOTICES.

The Physician's Visiting List for 1897. Forty-sixth year of its publication. Philadelphia: P. Blakiston, Son & Co., 1012 Walnut Street. Price for twenty-five patients per week, $1; fifty patients, $1.25; seventy-five patients, $2; one hundred patients, $2.25.

This list is the standard of the medical profession, and is used by nearly every physician of prominence in the neighborhood in which he practices. Some alterations have been made in its arrangement which have enhanced its value.

In the front pages there are some valuable reference tables and other items of information. Thus there is a table of the metric system of weights and measures, with tables for converting the English system into the French. There are elaborate dose tables, directions for resuscitation from asphyxia and apnœa, comparison of thermometers, and tables for calculating period of pregnancy. Apart from the daily record of visits there are pages for memoranda in general, for record of births; for addresses of patients, obstetric engagements, cash accounts and bills rendered. This same visiting list has been brought to the attention of the readers of this journal every year, and we do so again this year with pleasure.

